# Skin rash following Administration of Apalutamide in Japanese patients with Advanced Prostate Cancer: an integrated analysis of the phase 3 SPARTAN and TITAN studies and a phase 1 open-label study

**DOI:** 10.1186/s12894-020-00689-0

**Published:** 2020-09-02

**Authors:** Hiroji Uemura, Yosuke Koroki, Yuki Iwaki, Keiichiro Imanaka, Takeshi Kambara, Angela Lopez-Gitlitz, Andressa Smith, Hirotsugu Uemura

**Affiliations:** 1 Department of Urology and Renal Transplantation, Yokohama City University Medical Center, Yokohama, Japan; 2 Medical Affairs, Janssen Pharmaceutical K.K., Tokyo, Japan; 3 Clinical Pharmacology, Janssen Pharmaceutical K.K, Tokyo, Japan; 4 Clinical Science, Janssen Pharmaceutical K.K, Tokyo, Japan; 5 Department of Dermatology, Yokohama City University Medical Center, Yokohama, Japan; 6 Janssen Global Research & Development, Spring House, PA USA; 7 Department of Urology, Kindai University Faculty of Medicine, Osaka, Japan

**Keywords:** Apalutamide, Prostate cancer, Skin rash, Japanese

## Abstract

**Background:**

A higher incidence of apalutamide-related skin rash has been observed in Japanese patients with prostate cancer (PC).

**Methods:**

This integrated analysis of data of Japanese patients from 2 global Phase 3 studies, SPARTAN (NCT01946204; patients with non-metastatic castration-resistant PC [nmCRPC]) and TITAN (NCT02489318; patients with metastatic castration-sensitive PC [mCSPC]), and the Phase 1 study 56021927PCR1008 (NCT02162836; patients with metastatic CRPC [mCRPC]), assessed clinical risk factors of apalutamide-related skin rash as well as the potential correlation with plasma exposure to apalutamide. Kaplan-Meier method was used for time-to-event analyses. Clinical risk factors for skin rash were assessed using odds ratio.

**Results:**

Data from 68 patients (SPARTAN: *n* = 34, TITAN: *n* = 28, 56021927PCR1008: *n* = 6) receiving apalutamide 240 mg orally once-daily were analyzed. Rash (13 [19.1%]) and maculo-papular rash (11 [16.2%]) were the most frequently reported skin rash. All Grade and Grade 3 skin rash occurred in 35 (51.5%) and 10 (14.7%) patients, respectively. Most (85.7%) skin rash occurred within 4 months of apalutamide initiation and resolved in a median time of 1 month following the use of antihistamines, topical or systemic corticosteroids, with/without apalutamide dose interruptions/reductions. Median time-to-remission of first incidence of rash and maximum grade incidence of rash were 1.0 month (IQR: 0.36–1.81) and 1.0 month (IQR: 0.30–2.43), respectively. No significant clinical risk factors for the incidence of skin rash were observed. Areas under the curve (0–24 h) (AUC_0–24, ss_) at steady-state of plasma apalutamide concentration were numerically slightly higher in patients with skin rash than those without.

**Conclusions:**

No clinical risk factors for rash could be detected. There is a potential correlation between incidence of skin rash and plasma exposure to apalutamide. In general, apalutamide-related skin rash is easily managed, with appropriate treatment with or without dose adjustment.

**Trial registration:**

Retrospective pooled analysis of NCT01946204, NCT02489318, and NCT02162836.

## Background

Apalutamide, an oral non-steroidal, second-generation, selective inhibitor of the androgen receptor (AR), binds directly to the ligand-binding domain of the AR, thereby preventing AR nuclear translocation and AR-mediated transcription, which in turn induces tumour cell death [1, 2]. Apalutamide is FDA-approved for the treatment of non-metastatic castration-resistant prostate cancer (nmCRPC) and metastatic castration-sensitive prostate cancer (mCSPC), based on data from two pivotal Phase 3 trials, SPARTAN (NCT01946204) and TITAN (NCT02489318), respectively. In the SPARTAN study, addition of 240 mg apalutamide once-daily (QD) to ongoing androgen-deprivation therapy (ADT) showed a significant increase in metastasis-free survival (MFS) (> 2 years) when compared to placebo, with an overall maintenance of health-related quality of life. However, the incidence of skin rash was higher in the apalutamide group compared to placebo (23.8% vs 5.5%) [3]. Similarly, in the TITAN study, skin rash was more frequently observed in the apalutamide group compared to placebo (27.1% vs 8.5%), although significant improvements in the dual primary endpoints of overall survival (OS) and radiographic progression-free survival (rPFS) were reported [4]. Rash is a grouped term that includes macular rash, maculo-papular rash, butterfly rash, erythematous rash, generalized rash, papules, papular rash, pruritic rash, pustular rash, systemic lupus erythematosus rash, erythema multiforme, stomatitis, and urticaria among others. It is important to note that the most commonly observed apalutamide-related rash is not graded based on severity but on body surface area (BSA) involved. Therefore, to be defined as a Grade 3 rash, it must cover > 30% BSA, regardless of concomitant symptoms, if any.

Interestingly, the subgroup analysis of Japanese patients in both the SPARTAN and TITAN studies showed that a greater proportion of patients treated with apalutamide developed skin rash compared with placebo (SPARTAN subgroup: 56.0% vs 0.0%; TITAN subgroup: 50.0% vs 8.7%) [5], [Uemura et al., *International Journal of Urology*, submitted], with the incidence in Japanese patients being nearly double the incidence observed in the global populations of the two studies. In a Phase 1 trial (56021927PCR1008, referred hereafter as PCR1008) conducted to study the safety, efficacy and pharmacokinetic (PK) profile of apalutamide in Japanese patients with metastatic CRPC (mCRPC) (NCT02162836) [6], skin rash was observed in 2/6 (33.3%) patients. Taken together, these data suggest that apalutamide-related skin rash is more frequently observed in Japanese patients. However, the relationship between patient characteristics and apalutamide-related skin rash is not clear. Moreover, the clinical risk factors for apalutamide-related skin rash and the relationship between apalutamide exposure and skin rash remains to be elucidated.

Therefore, we conducted an integrated analysis of data from Japanese patients in the SPARTAN and TITAN studies, along with data from the PCR1008 study primarily to investigate the incidence rate, types, severity and management of skin rash in Japanese subpopulation. Data from patients who developed skin rash were further analyzed to assess if the grade of skin rash had a correlation with the extent of exposure to apalutamide. In addition, various clinical risk factors, such as Gleason score and previous therapy were evaluated vis-à-vis their contribution to the incidence of skin rash.

## Methods

A pooled analysis of data from Japanese patients who were administered apalutamide in two global Phase 3 studies, SPARTAN and TITAN, and a Phase 1 study (PCR1008) was conducted. The study protocols and informed consent forms for the original trials had been reviewed and approved by their respective Independent Ethics Committee or Institutional Review Board. Studies were conducted in accordance with ethical principles outlined in the Declaration of Helsinki and were consistent with International Conference of Harmonization, Good Clinical Practices guidelines, and applicable regulatory requirements. Patients or their legally acceptable representatives provided written informed consent before enrollment. The original trials (SPARTAN, TITAN and PCR1008) were conducted and funded by Janssen Research & Development, LLC and Janssen Pharmaceuticals K.K.

The study design for each of the 3 studies is briefly presented below:

### SPARTAN

A total of 1207 patients with nmCRPC, receiving ongoing ADT, were randomly assigned (2:1) to either apalutamide (240 mg, QD, orally) or matched placebo, and treated until disease progression, withdrawal of consent, unacceptable toxicity or death. The primary efficacy endpoint was MFS. Secondary efficacy endpoints were time to metastasis, PFS, time to symptomatic progression, time to initiation of cytotoxic chemotherapy and OS.

### TITAN

A total of 1052 patients with mCSPC were randomized (1:1) to receive apalutamide (240 mg, QD, orally) plus ADT or placebo plus ADT, in this double-blind, Phase 3 trial. The dual primary endpoints were rPFS and OS. Secondary efficacy endpoints were time to cytotoxic chemotherapy, pain progression, chronic opioid use, and skeletal-related event.

#### Population pharmacokinetic analysis in SPARTAN and TITAN

A population PK analyses were performed using all available drug concentration data from SPARTAN and TITAN studies. The population PK model for plasma concentrations of apalutamide and its active metabolite, N-desmethyl apalutamide, was developed using nonlinear mixed-effects modeling. Individual exposure metrics i.e., areas under the curve after 24 h at steady-state (AUC_0–24, ss_ [μg*h/mL]) of plasma concentration, for apalutamide and N-desmethyl apalutamide were derived using post-hoc estimates of the PK parameters. AUC_0–24, ss_ was calculated based on an average dose up to time of the first rash event considering possible dose modifications. The details of the population PK model was reported in a separate article [7].

### PCR1008

A total of 6 patients were included in this Phase 1, open-label, multi-center study to analyze the safety, tolerability, and PK profile of apalutamide (240 mg, QD, orally) in Japanese patients with mCRPC who were on ADT background therapy (either medical or surgical castration). Patients received a single apalutamide 240 mg dose on Day 1 of the PK week. Patients were reassessed for safety. If no safety signals were reported, patients received apalutamide 240 mg QD until disease progression, unacceptable toxicity, withdrawal of consent, or death, whichever occurred first. Safety was assessed. All adverse events (AEs) were graded according to National Cancer Institute Common Terminology Criteria for Adverse Events (NCI CTCAE), v4.0.3 in the SPARTAN and TITAN studies, while NCI CTCAE v4.0 was used in PCR1008.

#### Analysis

##### Incidence rate, type of rash, severity, management

The incidence rate, type and grade of rash, along with the treatments for the management of rash in each of the 3 studies was analyzed.

##### Time-to-event analysis

The time-to-event analysis for rash was performed using the Kaplan-Meier method. Kaplan-Meier curves were generated for time-to-rash (All Grade and Grade 3 or more) and time-to-remission (first incidence, All Grade; first incidence, Grade 3 or more; last incidence, All Grade; and last incidence, Grade 3 or more) using individual patient-level data.

##### Clinical risk factors

Odds ratio and 95% confidence interval was used to assess whether factors including age; height; weight; body mass index; Eastern Cooperative Oncology Group Performance Status (ECOG PS); time from initial diagnosis to first dose, Gleason score at initial diagnosis; baseline prostate-specific antigen (PSA), alkaline phosphatase (ALP), hemoglobin, or lactate dehydrogenase levels; and previous local treatment, first-generation antiandrogens, or chemotherapy were related to the incidence of rash.

Pooled data for baseline characteristics, treatment duration, dose reduction, dose interruption, dose discontinuation, incidence of rash and management of rash are presented using descriptive statistics.

##### Plasma exposure and incidence of rash

The relationships between the occurrence (by grade) of skin rash and the plasma exposure (AUC_0–24, ss_) to apalutamide or N-desmethyl apalutamide were explored using boxplots. All exposure analyses were done based on data from the SPARTAN and TITAN studies.

## Results

**Fig. 1:**
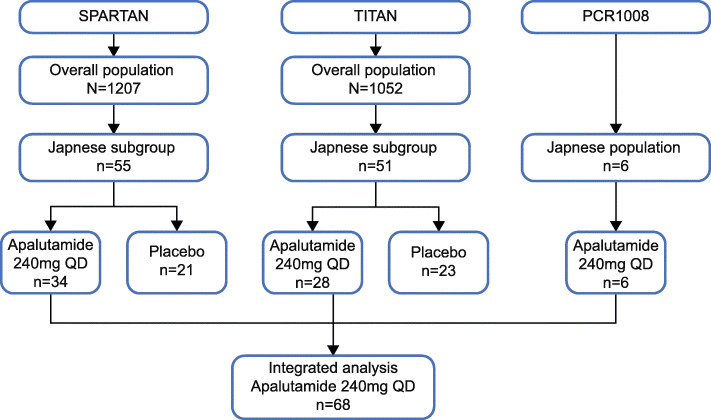
Flow diagram representing patient recruitment from SPARTAN, TITAN and PCR1008 studies

In total, 1207 patients in SPARTAN and 1052 patients in TITAN were enrolled globally, of which 55 and 51 patients were Japanese, respectively. This integrated analysis included data from total 68 Japanese patients (SPARTAN: *n* = 34, TITAN: *n* = 28, PCR1008: *n* = 6) who had received apalutamide 240 mg QD orally (Fig. 1).

### Baseline characteristics

Overall, the median age of patients was 77 years. Since the disease status at baseline was different in SPARTAN, TITAN, and PCR1008 studies, some of the baseline characteristics were not comparable between the three groups, with the median time from initial diagnosis, PSA levels, treatment durations, and ALP levels varying between the 3 patient populations. Median time from initial diagnosis to first dose was shortest in TITAN (2.19 months), followed by PCR1008 (68.89 months) and SPARTAN (87.10 months); with median PSA level [ng/mL] at baseline of 11.51, 54.42 and 4.35, respectively. All patients had received prior hormonal therapy (Table 1).

**Table 1:** Baseline Characteristics (Safety Set)

Categories	SPARTAN	TITAN	PCR1008	Total
**Number of patients in safety analysis set, n**	34	28	6	68
**Median age, years (range)**	79.00 (61–90)	73.00 (47–89)	78.00 (70–85)	77.00 (47–90)
**Median weight, kg (range)**	61.90 (45.5–84.0)	63.55 (40.6–89.2)	57.30 (49.8–62.6)	61.80 (40.6–89.2)
**Median height, cm (range)**^a^	163.50 (140.0–176.4)	165.20 (151.7–172.8)	163.55 (147.0–165.9)	164.00 (140.0–176.4)
**ECOG PS**^a^**, n (%)**				
0	30 (88.24)	25 (89.29)	5 (83.33)	60 (88.24)
1	4 (11.76)	3 (10.71)	1 (16.67)	8 (11.76)
**Median time from initial diagnosis to first dose, month (range)**	87.10 (11.01–176.10)	2.19 (1.28–74.15)	68.89 (9.23–132.72)	46.42 (1.28–176.10)
**GS at initial diagnosis, n (%)**				
≤ 7	9 (26.47)	1 (3.57)	2 (33.33)	12 (17.65)
≥ 8	25 (73.53)	27 (96.43)	4 (66.67)	56 (82.35)
**Median PSA at baseline, ng/mL (range)**	4.35 (0.45–21.71)	11.51 (0.04–457.64)	54.42 (8.92–310.11)	5.73 (0.04–457.64)
**Median Hemoglobin at baseline, ng/mL (range)**	12.95 (9.4–15.5)	13.45 (10.2–15.9)	11.95 (9.9–15.0)	13.10 (9.4–15.9)
**Median LDH at baseline, U/L (range)**	–	198.00 (123–272)	192.00 (115–456)	197.50 (115–456)
**Median ALP at baseline, U/L (range)**	73.50 (33–137)	104.50 (56–1149)	204.50 (102–331)	87.00 (33–1149)
**Disease status, n**				
nmCRPC	55	–	–	55
mCSPC	–	51	–	51
mCRPC	–	–	6	6
**Tumor volume**^b^**, n (%)**				
High	–	18 (64.29)	–	18 (64.29)
Low	–	10 (35.71)	–	10 (35.71)
**Previous treatment, n (%)**				
*Local treatment*	21 (61.76)	1 (3.57)	2 (33.33)	24 (35.29)
RP	5 (14.71)	1 (3.57)	1 (16.67)	7 (10.29)
RT	19 (55.88)	1 (3.57)	1 (16.67)	21 (30.88)
*Hormonal treatment*	34 (100.00)	28 (100.00)	6 (100.00)	68 (100.00)
LHRHa	34 (100.00)	28 (100.00)	5 (83.33)	67 (98.53)
Orchiectomy	1 (2.94)	2 (7.14)	1 (16.67)	4 (5.88)
1st generation AA	33 (97.06)	15 (53.57)	6 (100.00)	54 (79.41)
Other	2 (5.88)	0 (0.00)	0 (0.00)	2 (2.94)
*Chemotherapy*	0 (0.00)	0 (0.00)	0 (0.00)	0 (0.00)
*Other*	3 (8.82)	0 (0.00)	2 (33.33)	5 (7.35)
**Average dose of Apalutamide**				
**Mean dose, mg (SD)**	223.94 (28.91)	214.07 (44.90)	225.84 (25.76)	220.04 (36.05)
**Median dose, mg (range)**	236.55 (108.6–240.0)	239.02 (90.6–240.0)	236.10 (174.2–240.0)	238.27 (90.6–240.0)

*AA* Antiandrogen, *ALP* Alkaline phosphatase, *ECOG* Eastern Cooperative Oncology Group, *GS* Gleason score, *LDH* Lactate dehydrogenase LHRHa, hormone-releasing hormone antagonist, *nmCRPC* Non-metastatic castration resistant prostate cancer, *mCSPC* Metastatic castration sensitive prostate cancer, *mCRPC* Metastatic castration resistant prostate cancer, *PSA* Prostate-specific antigen, *RP* Radical prostatectomy, *RT* Radiotherapy, *SD* Standard deviation

^a^: count PCR1008 at screening

^b^: Denominator of proportion is TITAN’s safety analysis set

### Incidence rate, types of rash, severity

In the global SPARTAN and TITAN studies, the overall incidence of skin rash in the apalutamide group was 191/803 (23.8%) and 142/524 (27.1%), respectively, with the combined incidence rates of the most commonly reported rash in the 2 studies, i.e., rash, generalized rash, and maculo-papular rash, being 167/1327 (12.6%), 53/1327 (4.0%), and 60/1327 (4.5%), respectively (Supplementary Table 1). In the present integrated analysis of Japanese patients from SPARTAN and TITAN, and PCR1008, skin rash was observed in 35/68 (51.5%) of the patients, and the incidence rates of rash (13/68 [19.1%]), generalized rash (11/68 [16.2%]), and maculo-papular rash (11/68 [16.2%]) (Table 2) were also higher than that observed in the global studies. Also, the incidence rate of the less commonly observed rash, erythema multiforme and stomatitis, were higher in Japanese patients (3/68 [4.4%], each) compared to their combined incidence rates in the global SPARTAN and TITAN studies (6/1327 [0.45%] and 10/1327 [0.75%], respectively) (Supplementary Table 1).

**Table 2:** Types of Skin Rash in Apalutamide-treated Patients

Analysis Set (N = 68)	Total n (%)	Grade 1 n (%)	Grade 2 n (%)	Grade 3 n (%)
**Rash**	35 (51.47)	9 (13.23)	16 (23.52)	10 (14.70)
Rash	13 (19.11)	8 (11.76)	4 (5.88)	1 (1.47)
Rash maculo-papular	11 (16.17)	2 (2.94)	6 (8.2)	3 (4.41)
Rash generalised	11 (16.17)	1 (1.47)	7 (10.29)	3 (4.41)
Erythema multiforme	3 (4.41)	0	1 (1.47)	2 (2.94)
Stomatitis	3 (4.41)	1 (1.47)	2 (2.94)	0
Urticaria	2 (2.94)	2 (2.94)	0	0
Blister	1 (1.47)	1 (1.47)	0	0
Drug eruption	1 (1.47)	0	0	1 (1.47)
Rash macular	1 (1.47)	0	0	1 (1.47)
Skin erosion	1 (1.47)	1 (1.47)	0	0
Skin exfoliation	1 (1.47)	1 (1.47)	0	0

In the global SPARTAN and TITAN studies, the incidence of Grade 1 skin rash was 69/803 (8.6%) and 57/524 (10.9%), respectively, in the apalutamide group, while the corresponding values for Grade 2 events were 80/803 (10.0%) and 52/524 (9.9%), respectively (Supplementary Table 1). In the Japanese patients analyzed for this study, the incidence of Grade 1 rash was similar between the groups (SPARTAN: 4/34 [11.8%], TITAN: 4/28 [14.3%], PCR1008: 1/6 [16.7%]), with overall incidence being 9/68 (13.2%); Grade 2 rash were more frequently observed in the SPARTAN study (10/34 [29.4%]) compared to the TITAN (5/28 [17.9%]) and PCR1008 (1/6 [16.7%]) studies; while Grade 3 rash was observed only in the SPARTAN (5/34 [14.7%]) and TITAN (5/28 [17.9%]) studies. Overall, Grade 3 skin rash occurred in 10/68 (14.7%) patients in the current integrated analysis, compared to 42/803 (5.2%) and 33/524 (6.3%) patients in the SPARTAN and TITAN global studies, respectively. (Supplementary Table 1) There were no Grade 4 or 5 rashes due to the grading criteria used for reporting the types of rash observed.

### Management of Rash

All Japanese patients with skin rash (35/68 [51.1%]) received supportive medications; oral antihistamine was the most common (25/35 [71.4%]), followed by systemic and topical corticosteroids (18/35 [51.4%] and 15/35 [42.9%], respectively) (Table 3). In comparison, in the global SPARTAN and TITAN studies, antihistamines were required in 67/191 (35.1%) and 54/142 (38.0%) patients, systemic corticosteroids in 33/191 (17.3%) and 29/142 (20.4%) patients, while topical corticosteroids were administered in 65/191 (34.0) and 61/142 (43.0%) patients, respectively (Supplementary Table 2).

**Table 3:** Rash Management

Analysis Set (N = 68)	SPARTAN	TITAN	PCR1008	Total
**Number of patients in safety analysis set, n**	34	28	6	68
Rash, n (%)	19 (55.88)	14 (50.00)	2 (33.33)	35 (51.47)
**Patients who received supportive care for rash, n (%)**				
Oral antihistamine	15 (78.95)	10 (71.43)	0 (0.00)	25 (71.43)
Systemic corticosteroid	4 (21.05)	14 (100.00)	0 (0.00)	18 (51.43)
Topical corticosteroid	15 (78.95)	0 (0.00)	0 (0.00)	15 (42.86)
Drug interruption	11 (57.89)	6 (42.86)	1 (50.00)	18 (51.43)
Dose reduction	4 (21.05)	3 (21.43)	0 (0.00)	7 (20.00)
Drug discontinuation	3 (15.79)	2 (14.29)	0 (0.00)	5 (14.29)
Other	0 (0.00)	0 (0.00)	1 (50.00)	1 (2.86)

Drug interruptions and dose reductions were required in 18/35 (51.4%) and 7/35 (20.0%) patients, respectively, with treatment discontinuation required in 5/35 (14.3%) patients. Among patients receiving apalutamide who developed skin rash in the global SPARTAN study, dose interruptions, does reductions, and treatment discontinuations were reported in 55/191 (28.8%), 22/191 (11.5%), and 19/191 (9.9%), patients, respectively, while the corresponding values in the global population from the TITAN study were 44/142 (31.0%), 28/142 (19.7%), and 12/142 (8.5%) (Supplementary Table 2).

### Time-to-event analyses

**Fig. 2:**
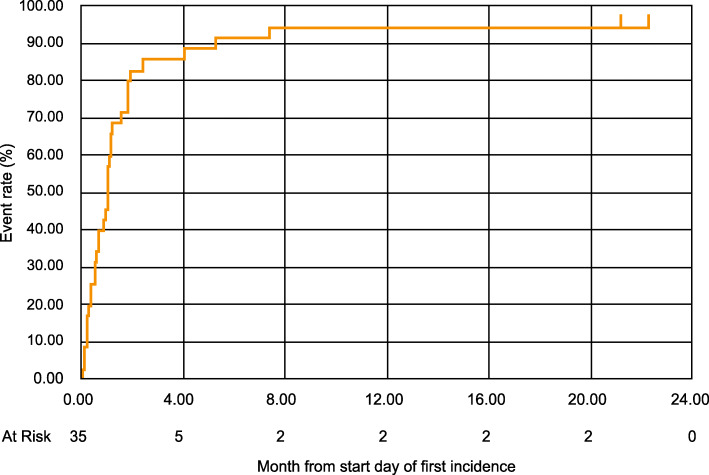
Kaplan-Meier plot for Time-to-Remission of First Incidence of rash (All Grade). Event: remission of first incidence of rash. Censor: not remission of first incidence of rash at the end of follow-up

**Fig. 3:**
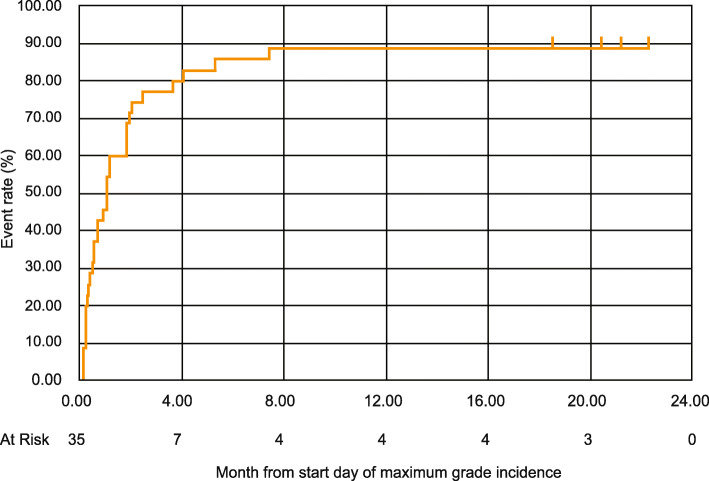
Kaplan-Meier plot for Time-to-Remission of Maximum Grade Incidence of rash (All grade). Event: remission of maximum Grade incidence of rash. Censor: not remission of maximum Grade incidence of rash at the end of follow-up

In the integrated analysis, the median time to onset of first incidence of rash of any grade in Japanese patients was 66 days, with time to incidence of first Grade 3 rash being 45 days and 9/10 (90.0%) Grade 3 events being reported in the first 4 months. The median time for first incidence of Grade 3 rash was 52 days and 38 days in the Japanese patients from SPARTAN and TITAN, respectively. In comparison, in the global population from the SPARTAN and TITAN studies, the median time to first incidence of skin rash were 82 days and 80.5 days, respectively (Supplementary Table 2). The median time for remission of first incidence of any grade in Japanese patients was 1.0 month (Interquartile range [IQR]: 0.36–1.81) (Fig. 2). In the global population of the SPARTAN study, skin rash of any grade resolved for 81% of the patients within 59.5 days, while the median time to resolution of skin rash of any grade in the TITAN study was 100 days (Supplementary Table 2). The time-to-remission of Grade 3 rash was 35 days and 17 days in the Japanese patients from SPARTAN and TITAN, respectively, with 1.0 month (IQR: 0.30–2.43) required for remission in the integrated analysis (Fig. 3). Some patients experienced skin rash more than once during apalutamide treatment. Even if patients resolved first incidence of rash, there was the potential for worsening in the second incidence of rash. Therefore, we assessed these two types of KM curves. KM figures for time-to-rash (All grade) (Supplementary Fig. 1), time-to-rash (≥Grade3) (Supplementary Fig. 2), time-to-remission of first incidence (≥Grade3) (Supplementary Fig. 3), and time-to-remission of maximum grade (≥Grade3) (Supplementary Fig. 4) are included in the supplementary files.

### Clinical risk factors

A number of clinical risk factors that could potentially affect the incidence of rash were assessed, including Eastern Cooperative Oncology Group Performance Status (ECOG PS), time from initial diagnosis to first dose, Gleason score, and previous treatments (Table 4). However, none of the factors were found to be significantly linked to the incidence of skin rash.

**Table 4:** Odds Ratio (All Grade), Target Population: Safety

Categories		Number of Patients	Rash, n (%)		95% CI	Odds Ratio	p-value
			Yes	No			
**Number of patients in safety analysis set, n**		68	35 (51.47)	33 (48.5)			
**Age class 1, year**	< 65	5	3 (60.00)	2 (40.00)			
	≥65	63	32 (50.79)	31 (49.20)	0.11–4.40	0.69	0.693
**Age class 2, year**	< 75	28	13 (46.43)	15 (53.57)			
	≥75	40	22 (55.00)	18 (45.00)	0.53–3.72	1.41	0.487
**Baseline weight**	< Median	34	16 (47.06)	18 (52.94)			
	≥ Median	34	19 (55.88)	15 (44.12)	0.55–3.70	1.43	0.467
**Baseline height**	< Median	34	15 (44.12)	19 (55.88)			
	≥ Median	34	20 (58.82)	14 (41.18)	0.69–4.73	1.81	0.227
**Body mass index (kg/m**^**2**^**)**	< 25	48	22 (45.83)	26 (54.17)			
	≥25	20	13 (65.00)	7 (35.00)	0.75–6.46	2.19	0.154
**ECOG PS**	0	60	30 (50.00)	30 (50.00)			
	1	8	5 (62.50)	3 (37.50)	0.37–7.61	1.67	0.510
**Time from initial diagnosis to first dose**	< Median	34	14 (41.18)	20 (58.82)			
	≥ Median	34	21 (61.76)	13 (38.24)	0.87–6.10	2.31	0.092
**GS at initial diagnosis**	≤ 7	12	9 (75.00)	3 (25.00)			
	≥ 8	56	26 (46.43)	30 (53.57)	0.07–1.18	0.29	0.084
	Unknown	0	0	0	–	–	
**Baseline PSA**	< Median	34	21 (61.76)	13 (38.24)			
	≥ Median	34	14 (41.18)	20 (58.82)	0.16–1.15	0.43	0.092
**Baseline Hemoglobin**	High	68	35 (51.47)	33 (48.53)			
	Normal or Low	0	0	0	–	–	–
**Baseline LDH**	High	7	2 (28.57)	5 (71.43)			0.282
	Normal or Low	27	14 (51.85)	13 (48.15)	0.44–16.37	2.69	
**Baseline ALP**	High	13	4 (30.77)	9 (69.23)			
	Normal or Low	55	31 (56.36)	24 (43.64)	0.80–10.59	2.91	0.106
**Previous local treatment**	No	44	21 (47.73)	23 (52.27)			
	Yes	24	14 (58.33)	10 (41.67)	0.56–4.19	1.53	0.404
**Previous 1st generation AA**	No	14	9 (64.29)	5 (35.71)			
	Yes	54	26 (48.15)	28 (51.85)	0.15–1.74	0.52	0.286
**Previous chemotherapy**	No	68	35 (51.47)	33 (48.53)			
	Yes	0	0	0	–	–	–

*AA* Antiandrogen, *ALP* Alkaline phosphatase, *ECOG PS* Eastern Cooperative Oncology Group Performance Status, *GS* Gleason score, *LDH* Lactate dehydrogenase, *PSA* Prostate-specific antigen, *CI* Confidence interval

### Pharmacokinetic analysis

**Fig. 4:**
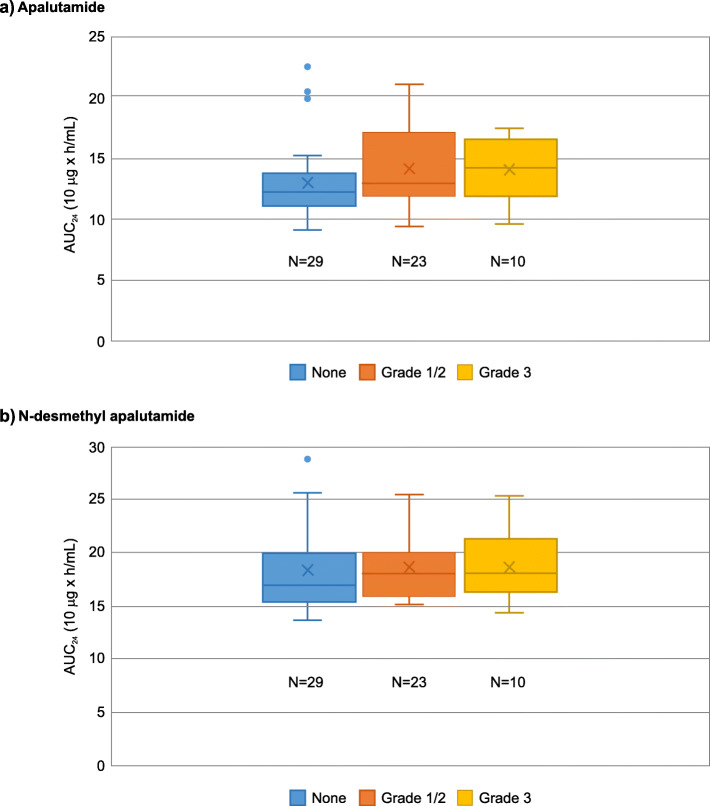
Plasma-Exposure and Incidence of Rash: Pooled results of Japanese patients from SPARTAN and TITAN studies. **a**) Apalutamide, **b**) N-desmethyl apalutamide. Relationships between incidence of rash (by grade) and the plasma exposure (AUC_0–24, ss_) to apalutamide or N-desmethyl apalutamide were explored using boxplots

AUC_0–24, ss_ of apalutamide were numerically slightly higher in patients with skin rash than those without; however, this did not significantly impact the grade of skin rash (Fig. 4a). No correlation was apparent with AUC_0–24, ss_ of N-desmethyl apalutamide (Fig. 4b).

## Discussion

Apalutamide-related skin rash has been observed in previous studies, with a higher incidence of skin rash observed in the Japanese subpopulation compared to overall global population. In the global SPARTAN and TITAN studies, and the Japanese subpopulation analysis from these studies [5], [Uemura et al., *International Journal of Urology*, submitted], Grade 3 rash (covering > 30% of the body surface) was observed more frequently with apalutamide [3, 4]. In the current integrated analysis, the incidence of Grade 3 rash (14.7%) was also higher than in the global SPARTAN and TITAN studies (combined incidence, 5.8%). This suggests that both the frequency and grade of skin rash, across different disease status, are higher in Japanese patients compared to the global population with apalutamide treatment. Consequently, treatment discontinuation due to rash, which is recommended if oral corticosteroids are required for > 28 days, was required in 14.3% patients in the current integrated analysis compared with 2.3% patients in the global population [3, 4]. Most Grade 1/2 rash resolved following the use of oral antihistamines, topical or systemic corticosteroids, with or without apalutamide dose interruption or reduction, following which, patients were able to continue apalutamide treatment. Based on the current analysis, it is difficult to conclude whether supportive treatment or dose interruption was more effective in resolving the different grades of skin rash.

In our study, the median time to first incidence of rash was 66 days, and it resolved in a median time of 1.0 month. When compared to SPARTAN, both the onset and resolution of rash were faster in Japanese patients. These data require further investigation since the overall incidence of skin rashes was higher in the Japanese patients, yet their time to incidence and resolution was faster. Interestingly, in our study, the median time to first remission was 1.0 month, but the median time-to-remission of last incidence was 3.6 months, suggesting that some patients experienced several incidents of skin rash. However, the median time-to-remission of the worst grade (Grade 3) of skin rash was 1.0 month. Therefore, even if some patients experienced several incidents of skin rash, the worst grade of skin rash remitted within 1.0 month following appropriate management by physicians. This could be attributed to the more frequent use of oral antihistamines (71.4%) and systemic corticosteroids (51.4%) as supportive medication among Japanese patients when compared with patients in the global studies (combined data from SPARTAN and TITAN: antihistamines, 36.5%; systemic corticosteroids, 18.5%).

Apalutamide and N-desmethyl apalutamide are two major pharmacologically active components detected in the systemic circulation at steady-state [8, 9]. The current integrated analysis showed that there was a possible correlation between plasma exposure to apalutamide and incidence of skin rash, but it did not significantly impact the grade of skin rash. However, the results should be interpreted with caution due to the limited sample size. The exposure-response modeling analysis for SPARTAN study based on whole study population was reported in a separate article [10].

Recently, the National Comprehensive Cancer Network (NCCN) guidelines were updated to recommend apalutamide or enzalutamide, another second-generation antiandrogen, in patients with nmCRPC with background ADT therapy [11]. Skin rash was not observed to be an adverse drug reaction (ADR) in Phase 3 trials, either in nmCRPC or mCRPC patients [12, 13] with enzalutamide, although a case report of enzalutamide-induced acute generalized exanthematous pustulosis was recently reported and the USPI includes rash as an ADR observed in post-marketing [14]. Skin rash has also been reported with the first-generation oral non-steroidal antiandrogen drug bicalutamide and other new-generation oral antiandrogen drugs such as darolutamide [15–18]. It remains to be elucidated if skin rash is a class-effect, particularly in Japanese patients.

There is evidence to suggest that ethnic difference and genotypic heterogeneity between the Japanese and Caucasian populations could have an impact on treatment responses in patients with PC [19–21]. The correlation of human leukocyte antigen (HLA) typing with serious drug-induced skin rash e.g., Stevens-Johnson syndrome or toxic epidermal necrosis, is well established. However, further studies to explore the correlation of HLA typing with apalutamide-related skin rash are needed. Interestingly, Japanese men have been observed to respond better to hormonal therapy compared to their Caucasian counterparts [22, 23]. With reports of skin rashes being disproportionately higher in the Japanese subset of SPARTAN and TITAN studies and no clinical risk factors identified in the current study, further studies are needed to understand the basis of the high incidence observed.

To assess the clinical risk factors associated with commonly administered PC drugs, an analysis of AEs among nmCRPC patients treated with abiraterone (a synthetic, steroidal CYP17A1 inhibitor recently approved in the treatment of PC), enzalutamide, or bicalutamide, was carried out in the real-world setting. Baseline AEs, Charlson comorbidity index, surgical castration, and older age were found to be significant predictors of AEs [24]. However, in our study, which was conducted based on registration trials, no clinical risk factors of skin rash were identified following apalutamide treatment. Therefore, it is important to analyze clinical risk factors of skin rash using real-world evidence.

A recent study assessed important safety factors that physicians considered in making treatment decisions for patients with nmCRPC. Among the safety attributes analyzed, which included incidence of skin rash, physicians were most concerned regarding cognitive problems, fractures, and fatigue. In fact, physicians regarded reduction in cognitive problems (from severe to none) to have a 36.0% higher importance in comparison to improving OS by 12 months instead of 3 months. Therefore, even though an important adverse event, skin rash was not a major determinant in physicians’ choice of treatment for PC, reiterating the need to assess the benefit-risk ratio to determine the best treatment for a particular patient with PC [24, 25].

Some of the baseline characteristics were not comparable between SPARTAN, TITAN and PCR1008 groups, with the median time from initial diagnosis, PSA levels, treatment durations, and ALP levels varying between these patient populations, since these were inherently different patient populations with different disease status at baseline. Although the cohorts were relatively dissimilar, the overall apalutamide regimens were similar amongst different disease populations in three studies. There may be an observer bias in an open-label Phase 1 study, however, it was included in the pooled analysis since the treatment dose (240 mg/day) and the incidence of skin rash were similar to those in Phase 3 studies. In current research, we focussed on Japanese subpopulation for incidence of apalutamide-related skin rash. Most of skin rash occurred in the first 4 months after apalutamide initiation and it would be difficult to evaluate time to skin rash stratified by covariates since the patients’ number is small. In order to conduct time-to-event analysis, larger apalutamide treatment data is warranted in the real-world settings.

The generalizability of the findings in this study is limited by the small number of patients analyzed (*N* = 68), its unplanned retrospective design, and the fact that the integrated analysis was done on different disease populations (nmCRPC, mCSPC and mCRPC). However, the results of this study underscore the need to undertake a prospective analysis of apalutamide-related skin rash in the Japanese population.

## Conclusions

The incidence of apalutamide-related skin rash was higher in Japanese patients with PC compared to patients from the rest of the world. Although there is a potential correlation between incidence of skin rash and plasma exposure to apalutamide, it did not significantly impact the grade of skin rash. Moreover, no clinical risk factors associated with skin rash were identified. Most skin rash is observed within 120 days of treatment initiation and close monitoring of the skin rash, dose reductions/interruptions and treatment with oral antihistamines and topical and systemic corticosteroids led to resolution of the majority of skin rash observed in Japanese patients within 30 days.

## Supplementary information

**Additional file 1.** **Supplementary Fig. 1.** Kaplan-Meier plot for Time-to-Rash (All Grade).

**Additional file 2.** **Supplementary Fig. 2.** Kaplan-Meier plot for Time-to-Rash (≥ Grade 3).

**Additional file 3.** **Supplementary Fig. 3.** Kaplan-Meier plot for Time-to-Remission of First Incidence of rash (≥ Grade 3).

**Additional file 4.** **Supplementary Fig. 4.** Kaplan-Meier plot for Time-to-Remission of Maximum Grade Incidence of rash (≥ Grade 3).

**Additional file 5.** **Supplementary Table 1.** Incidence of Rash in SPARTAN and TITAN.

**Additional file 6.** **Supplementary Table 2.** Time to Incidence of Rash, Rash Management, and Resolution of Rash in SPARTAN and TITAN.

## Data Availability

The study materials and datasets used and/or analyzed during the current study are available from the corresponding author on reasonable request.

## Abbreviations

ADR: Adverse drug reaction
ADT: Androgen-deprivation therapy
ALP: Alkaline phosphatase
AR: Androgen receptor
BSA: Body surface area
CI: Confidence interval
ECOG PS: Eastern Cooperative Oncology Group Performance Status
HLA: Human leukocyte antigen
mCSPC: Metastatic castration-sensitive prostate cancer
mCRPC: Metastatic castration-resistant prostate cancer
MFS: Metastasis-free survival
NCCN: National Comprehensive Cancer Network
NCI CTCAE: National Cancer Institute Common Terminology Criteria for Adverse Events
nmCRPC: Non-metastatic castration-resistant prostate cancer
OS: Overall survival
PFS: Progression-free survival
PK: Pharmacokinetic
PSA: Prostate-specific antigen
QD: Once-daily
SD: Standard deviation
