# Correction to: Skin rash following Administration of Apalutamide in Japanese patients with Advanced Prostate Cancer: an integrated analysis of the phase 3 SPARTAN and TITAN studies and a phase 1 open-label study

**DOI:** 10.1186/s12894-020-00739-7

**Published:** 2020-10-22

**Authors:** Hiroji Uemura, Yosuke Koroki, Yuki Iwaki, Keiichiro Imanaka, Takeshi Kambara, Angela Lopez-Gitlitz, Andressa Smith, Hirotsugu Uemura

**Affiliations:** 1 Department of Urology and Renal Transplantation, Yokohama City University Medical Center, Yokohama, Japan; 2 Medical Affairs, Janssen Pharmaceutical K.K., Tokyo, Japan; 3 Clinical Pharmacology, Janssen Pharmaceutical K.K, Tokyo, Japan; 4 Clinical Science, Janssen Pharmaceutical K.K, Tokyo, Japan; 5 Department of Dermatology, Yokohama City University Medical Center, Yokohama, Japan; 6 Janssen Global Research & Development, Spring House, PA USA; 7 Department of Urology, Faculty of Medicine, Kindai University, Osaka, Japan

## Correction to: BMC Urol (2020) 20:139 https://doi.org/10.1186/s12894-020-00689-0

In the original publication of this article [1] there were several errors in Table 1 related to the values for Disease status (nmCRPC and mCSPC).

In this correction article the correct and incorrect values are shown.

**Table 1** The correct and incorrect values

**Table:** 

Incorrect					Correct				
Disease status, n					Disease status, n				
nmCRPC	55	–	–	55	nmCRPC	34	–	–	34
mCSPC	–	51	–	51	mCSPC	–	28	–	28

Furthermore, the **Time-to-event analyses** section has several errors with the decimal values/rounding of numbers, the incorrect and correct information is shown below

**Table:** 

Incorrect	Correct
66 days	66.0 days
45 days	45.0 days
52 days	52.0 days
38 days	38.0 days
82 days	82.0 days
In the global population of the SPARTAN study, skin rash of any grade resolved for **81%** of the patients within 59.5 days, while the median time to resolution of skin rash of any grade in the TITAN study was 100 days (Supplementary Table 2)	In the global population of the SPARTAN study, skin rash of any grade resolved for **80.6%** of the patients within 59.5 days, while the median time to resolution of skin rash of any grade in the TITAN study was 100.0 days (Supplementary Table 2)
100 days	100.0 days
35 days	35.0 days
37 days	37.0 days
66 days	66.0 days
