# Supplementary material for: Skin rash following Administration of Apalutamide in Japanese patients with Advanced Prostate Cancer: an integrated analysis of the phase 3 SPARTAN and TITAN studies and a phase 1 open-label study
Source: BMC Urol. 2020 Sep 2;20:139. doi: 10.1186/s12894-020-00689-0 (PMC7465330; doi:10.1186/s12894-020-00689-0)
Supplement: Supplementary file 5 — Additional file 5. Supplementary Table 1. Incidence of Rash in SPARTAN and TITAN. [file 12894_2020_689_MOESM5_ESM.docx]

**Supplementary Table 1: Incidence of Rash in SPARTAN and TITAN**

|  | **SPARTAN** | | | | **TITAN** | | | | **Combined Incidence** |
| --- | --- | --- | --- | --- | --- | --- | --- | --- | --- |
|  | **Total** | **Grade 1** | **Grade 2** | **Grade 3** | **Total** | **Grade 1** | **Grade 2** | **Grade 3** |  |
| **Number of patients who received apalutamide in safety analysis set, n** | 803 |  |  |  | 524 |  |  |  | 1327 |
| **Skin Rash, n (%)** | 191 (23.8) | 69 (8.6) | 80 (10.0) | 42 (5.2) | 142 (27.1) | 57 (10.9) | 52 (9.9) | 33 (6.3) | 333 (25.1) |
| Rash | 87 (10.8) | 44 (5.5) | 33 (4.1) | 10 (1.2) | 80 (15.3) | 49 (9.4) | 25 (4.8) | 6 (1.1) | 167 (12.6) |
| Rash maculo-papular | 43 (5.4) | 10 (1.2) | 18 (2.2) | 15 (1.9) | 17 (3.2) | 3 (0.6) | 6 (1.1) | 8 (1.5) | 60 (4.5) |
| Rash generalized | 19 (2.4) | 3 (0.4) | 9 (1.1) | 7 (0.9) | 34 (6.5) | 7 (1.3) | 13 (2.5) | 14 (2.7) | 53 (4.0) |
| Erythema multiforme | 4 (0.5) | 0 | 2 (0.2) | 2 (0.2) | 2 (0.4) | 0 | 0 | 2 (0.4) | 6 (0.45) |
| Stomatitis | 3 (0.4) | 3 (0.4) | 0 | 0 | 7 (1.3) | 4 (0.8) | 3 (0.6) | 0 | 10 (0.75) |
| Urticaria | 16 (2.0) | 6 (0.7) | 8 (1.0) | 2 (0.2) | 4 (0.8) | 3 (0.6) | 1 (0.2) | 0 | 20 (1.5) |
| Blister | 1 (0.1) | 1 (0.1) | 0 | 0 | 3 (0.6) | 3 (0.6) | 0 | 0 | 4 (0.30) |
| Drug eruption | 2 (0.2) | 0 | 1 (0.1) | 1 (0.1) | 2 (0.4) | 0 | 0 | 2 (0.4) | 4 (0.30) |
| Rash macular | 10 (1.2) | 1 (0.1) | 4 (0.5) | 5 (0.6) | 1 (0.2) | 0 | 0 | 1 (0.2) | 11 (0.83) |
| Skin erosion | 1 (0.1) | 1 (0.1) | 0 | 0 | 0 | 0 | 0 | 0 | 1 (0.07) |
| Skin exfoliation | 4 (0.5) | 4 (0.5) | 0 | 0 | 5 (1.0) | 4 (0.8) | 1 (0.2) | 0 | 9 (0.68) |
