# Supplementary material for: Skin rash following Administration of Apalutamide in Japanese patients with Advanced Prostate Cancer: an integrated analysis of the phase 3 SPARTAN and TITAN studies and a phase 1 open-label study
Source: BMC Urol. 2020 Sep 2;20:139. doi: 10.1186/s12894-020-00689-0 (PMC7465330; doi:10.1186/s12894-020-00689-0)
Supplement: Supplementary file 6 — Additional file 6. Supplementary Table 2. Time to Incidence of Rash, Rash Management, and Resolution of Rash in SPARTAN and TITAN. [file 12894_2020_689_MOESM6_ESM.doc]

**Supplementary Table 2: Time to Incidence of Rash, Rash Management, and Resolution of Rash in SPARTAN and TITAN**

| **Categories** | **SPARTAN** | **TITAN** |
| --- | --- | --- |
| **Number of patients who received apalutamide in safety analysis set,** n | 803 | 524 |
| **Patients with skin rash,** n | 191 | 142 |
| **Median time to onset of first rash,** days | 82.0 days | 80.5 days |
| **Patients who received supportive care for rash** |  |  |
| Oral antihistamine, n (%) | 67 (35.1) | 54 (38.0) |
| Systemic corticosteroid, n (%) | 33 (17.3) | 29 (20.4) |
| Topical corticosteroid, n (%) | 65 (34.0) | 61 (43.0) |
| Drug interruption, n (%) | 55 (28.8) | 44 (31.0) |
| Dose reduction, n (%) | 22 (11.5) | 28 (19.7) |
| Drug discontinuation, n (%) | 19 (9.9) | 12 (8.5) |
| **Median time to resolution of skin rash of any grade**, days (% of patients) | 59.5 days (80.6% patients) | 100.0 days (75.4% patients) |
